# Unveiling the Human Brain Virome in Brodmann Area 46: Novel Insights Into Dysbiosis and Its Association With Schizophrenia

**DOI:** 10.1093/schizbullopen/sgad029

**Published:** 2023-10-12

**Authors:** Mahin Ghorbani

**Affiliations:** Department of Dental Medicine, Division of Oral Diagnostics and Rehabilitation, Karolinska Institute, Stockholm, Sweden; Department of Laboratory Medicine, Division of Pathology, Karolinska Institute, Stockholm, Sweden

**Keywords:** metavirome, whole-genome sequence (WGS), viruses, mental disorders, psychosis, viral infection

## Abstract

Research suggests a potential role of the oral-neuro and gut-brain axes in schizophrenia, involving non-brain microbiomes such as salivary and gut microbiomes. However, the blood-brain barrier effectively prevents microorganism entry. Additionally, despite approximately 8% of the human genome consisting of *retroviruses* and the established link between viral infections and schizophrenia, the presence of a resident virome (a viral component of the microbiome) in the brain and its association with mental disorders remain unexplored. Methods: Whole-genome sequencing raw data from postmortem Brodmann Area 46 (BA46) tissue from 49 individuals (20 healthy controls [HCs], 29 with schizophrenia [SCZs]) obtained from the NCBI SRA database from BioProject: PRJNA422380.Virome profiles were retrieved using Metaphlan3, and viral signatures were identified using linear discriminant analysis effect size (LEfSe). Mann-Whitney tests and receiver operating characteristic curve validated the viral signatures. Results: In BA46, 30 distinct species representing 9 phyla, 10 classes, 10 orders, 13 families, and 19 genera were identified. HCs exhibited greater alpha diversity, and there were significant differences in beta diversity between the groups. LEfSe analysis highlighted distinct viral levels, including *Escherichia virus Lambda*, *Escherichia virus phiV10*, *Human endogenous retrovirus K*, *Taterapox virus*, *Alcelaphine gammaherpesvirus 1*, and *Bovine gammaherpesvirus 4* in HCs, while *Glypta fumiferanae ichnovirus* and unknown virus showed higher levels in schizophrenia. Conclusion: This is the first study to identify a human brain virome associated with schizophrenia in BA46. Brain virome dysbiosis may be associated with mental illness, and viral signatures may serve as biomarkers for the early detection of schizophrenia.

## Introduction

Schizophrenia is a severe and persistent mental illness. According to the World Health Organization (WHO), approximately 24 million people worldwide are affected by schizophrenia. This complex disorder is characterized by various symptoms, including delusions, hallucinations, abnormal thinking and behavior, and emotional blunting.^1^ Despite extensive research efforts, the exact origins of schizophrenia remain unknown. However, evidence suggests that a combination of genetic, environmental, neurological, viral, and immunological factors may contribute to its development.^2–4^ While investigations have been conducted to explore the potential involvement of viruses in the onset of schizophrenia, their precise role in the disease remains unclear. Several studies have focused on the association between viral infections and schizophrenia, specifically examining viruses such as *herpes simplex virus type 1* (*HSV-1*), *cytomegalovirus* (*CMV*), and *influenza virus*.^5–8^ One possible mechanism that has been proposed is neurodevelopmental disruption, which suggests that viral infections during critical periods of brain development can interfere with the normal maturation of neural circuits. This disruption may increase the susceptibility to psychiatric disorders, including schizophrenia. Previous studies have reported an elevated risk of schizophrenia in individuals who were exposed to certain viral infections either during prenatal development or early childhood.^9,10^ Moreover, research has also explored the potential interaction between genetic susceptibility and viral infections in mental disorders such as schizophrenia. It is believed that individuals with a genetic predisposition to schizophrenia may be more susceptible to the effects of viral infections, and specific genetic variations related to immune response and synaptic function have been identified as potential factors contributing to this interaction.^11,12^ Furthermore, neuroimmune dysregulation has been proposed as a potential link connecting viral infections and schizophrenia. Viral infections have the potential to trigger the immune system, resulting in the release of pro-inflammatory cytokines and various other immune molecules. Excessive or prolonged immune activation during critical periods of brain development may disrupt neural circuits and contribute to the development of schizophrenia.^13–15^ It is essential to note that while these studies suggest a potential association between viral infections and schizophrenia, they do not establish a causal relationship. Schizophrenia is a complex disorder with multifactorial origins, and viral infections are likely just one of many contributing factors. Further research is necessary to gain a better understanding of the precise mechanisms underlying this association and to determine the extent of the viral contribution to the development of schizophrenia. Recently, scientists have begun to explore the role of non-brain localized microbiomes such as oral and gut microbiomes in the etiology of schizophrenia.^16–18^ The microbiome refers to the complex ecology of microorganisms naturally present within and on the human body. While most studies have focused on the bacterial component of the microbiome, the viral diversity within the microbiome, known as the virome, remains less understood, particularly in the context of neuropsychiatric disorders. Despite extensive evidence of the presence of 8% of endogenous *retroviruses* in human genomes and the importance of viral infections in schizophrenia,^19–21^ there has been no research into the presence of the brain virome, which may contribute to brain illnesses through virome dysbiosis. This study hypothesizes the existence of a resident virome in the brain, which refers to the brain virome that has coevolved with humans and persists even when it is not actively replicating. The diversity and balanced composition of this virome are believed to be essential for maintaining a healthy brain. Unlike conditions such as brain abscesses or encephalopathies, which are characterized by the proliferation of pathogenic microbes, this hypothesis suggests the presence of nonpathogenic brain viruses that may contribute to imbalances and dysbiosis, ultimately affecting brain health and disease. To test this hypothesis, this study aims to investigate the presence of a virome profile in Brodmann Area 46 (BA46), also known as the dorsolateral prefrontal cortex (DLPFC), a specific brain region located in the frontal lobe. BA46 area plays a crucial role in higher cognitive functions such as working memory, attention, decision-making, and executive control.^22,23^ Consequently, there have been numerous studies investigating the potential involvement of BA46/DLPFC abnormalities in schizophrenia.^24–26^

Therefore, the primary objective of this study is to investigate the presence of a brain virome and the potential role of virome dysbiosis in schizophrenia, specifically within the BA46. Identifying viral components of the microbiome that may contribute to dysbiosis, and understanding their role in the development of schizophrenia, could pave the way for new treatments and preventive measures.

## Materials and Methods

### Data Collection

In this study, raw brain sample data were obtained from the NCBI SRA database (https://www.ncbi.nlm.nih.gov/sra) from BioProject: PRJNA422380 in which scientists analyzed methylome and transcriptome data^27^ and found significant insights into the epigenetic dysregulation associated with schizophrenia. For the current study, a total of 49 samples were downloaded, including 20 healthy controls (HCs) and 29 schizophrenia (SCZs), matched age and gender (Supplementary Table 1) provides information regarding the selected samples, encompassing data such as NCBI accession numbers, age, gender, and read length.

### Bioinformatics and Statistical Analysis

FastQC v0.11.8 was used to verify the quality of raw RNA-Seq data. Cutadapt v2.8 was used to eliminate adaptor sequences and low-quality bases from raw data. The preprocessed sequencing data were analyzed by MetaPhlAn3,^28^ which relies on unique clade-specific marker genes discovered from 17 000 reference genomes (13 500 bacterial and archaeal, 3500 viral, and 110 eukaryotic taxa). To filter out bacterial, eukaryotic, and archaeal taxa, the commands “--exclude bacteria,” “--exclude eukaryotes,” and “--exclude archaea” were applied. Taxonomic assignments were made using the in-house MetaPhlAn3 database. The feature count table was filtered to eliminate counts >2 with a sample prevalence >10%. The feature count table used for subsequent analysis underwent preparation through total-sum scaling normalization, followed by rarefaction to ensure sample depth normalization. Alpha diversity metrics such as Observed, Chao1, Shannon, Simpson, as well as differential viral communities (beta diversity) between HCs and SCZs using the Bray-Curtis and Jaccard index distances based on nonmetric multidimensional scaling (NMDS) and the PERMANOVA significance test, were calculated in R using the vegan package v2.5.6. Linear discriminant analysis effect size (LEfSe v1.1.01) (LDA score >2, and *P* < .05),^29^ was used to detect differentially abundant viral species between HCs and SCZs. The receiver operating characteristic (ROC) analysis was used to estimate the predictive value of each discovered viral species. Spearman correlation was used for correlation analysis. Heatmaps of the core virome were created in Microbiome Analyst server.^30^ The study used CombiROC, a method that integrates numerous markers, to discover the best viral species combination for discriminating between HCs and SCZs.^31^

## Results

### Study Design and Participants

**Fig. 1.**
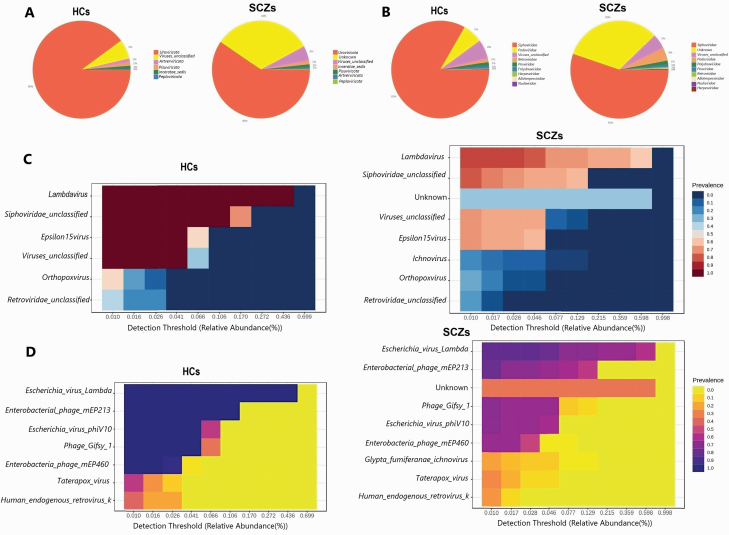
Brain virome composition profiles and core brain virome. (A) Pie chart depicting the viral distribution at the phylum level in HCs and SCZs. (B) Pie chart illustrating the family-level viral distribution of in HCs and SCZs. (C) Heatmap of the core virome of HCs and SCZs at the genus level. (D) Heatmap of the core virome of HCs and SCZs at the species level. HCs, healthy controls; SCZs, schizophrenia.

In total, 11054880264 sequence reads were collected from 49 samples, with a range of 42858676 to 513859759 and an average of 225609801 reads. After applying a 10% prevalence threshold and a minimum read count of 2 for filtration, 91 virus types were recovered and assigned to 9 phyla, 10 classes, 10 orders, 13 families, 19 genera, and 30 species. *Uroviricota* was the dominant phylum in both groups (HCs: 90%, SCZs: 60%). Notably, the SCZs exhibited a 30% reduction in the abundance of *Uroviricota* compared with the HCs, as depicted in an interactive pie chart. Furthermore, the SCZs presented a 32% prevalence of unknown viruses at the phylum level, a feature was absent in HCs. Both groups included *unclassified viruses*, *incertae sedis*, *Pisuviricota*, *Artverviricota*, and *Peploviricota* in their phylum profiles, each accounting for minor percentages ranging from 0.24% to 6.4% (figure 1A).

At the family level, the interactive pie chart revealed that *Siphoviridae* was the most abundant family in both groups (HCs: 83%, SCZs: 55%). In comparison to the HCs, the prevalence of *Siphoviridae* was 28% lower in the SCZs. Furthermore, SCZs exhibited a 32% prevalence of unknown viruses at the family level, which were not detected in HCs. Both groups displayed *Podoviridae*, *unclassified viruses*, *Retroviridae*, *Poxviridae*, and *Polydnaviridae* at minor percentages in their profiles, ranging from 0.89% to 6.6% (figure 1B). To establish core viromes, a prevalence threshold of 20% was set in each group, with a minimum abundance of 0.01% (based on the minimum cutoff of the Microbiome Analyst server). The core virome of HCs consisted of 6 genera and 7 species of taxa, while the core virome of SCZs included 8 genera and 9 species of taxa. The most prevalent genera in both groups were *Lambdavirus*, *Siphoviridae unclassified*, *unclassified viruses*, and *Epsilon15virus*, with 100% prevalence in HCs and 83%, 79%, 69%, and 69% prevalence in SCZs, respectively. Furthermore, in SCZs, an unknown virus was predominant, accounting for 34% prevalence. Additionally, *Orthopoxvirus* (HCs: 55%, SCZs: 28%) and *Retroviridae unclassified* (HCs: 40%, SCZs: 24%) were the most abundant genera in both groups (figure 1C). Analyzing the dominant core virome at the species level through heatmaps revealed that the most prevalent species included *Escherichia virus Lambda*, *Enterobacterial phage mEp213*, *Phage Gifsy 1*, *Escherichia virus phiV10*, and *Enterobacteria phage mEp460*, with 100% prevalence in HCs and 83%, 79%, 69%, 69%, and 66% prevalence in SCZs, respectively. Furthermore, the SCZs exhibited a 34% prevalence of unknown viruses, which were absent in HCs. *Taterapox virus*, *Human endogenous retrovirus K*, and *Glypta fumiferanae ichnovirus* were among the most prevalent viruses in both groups (HCs: 55%, 40%, 20%; SCZs: 28%, 24%, 20%) (figure 1D).

### Virome Richness and Diversity in Participants’ Brains

**Fig. 2.**
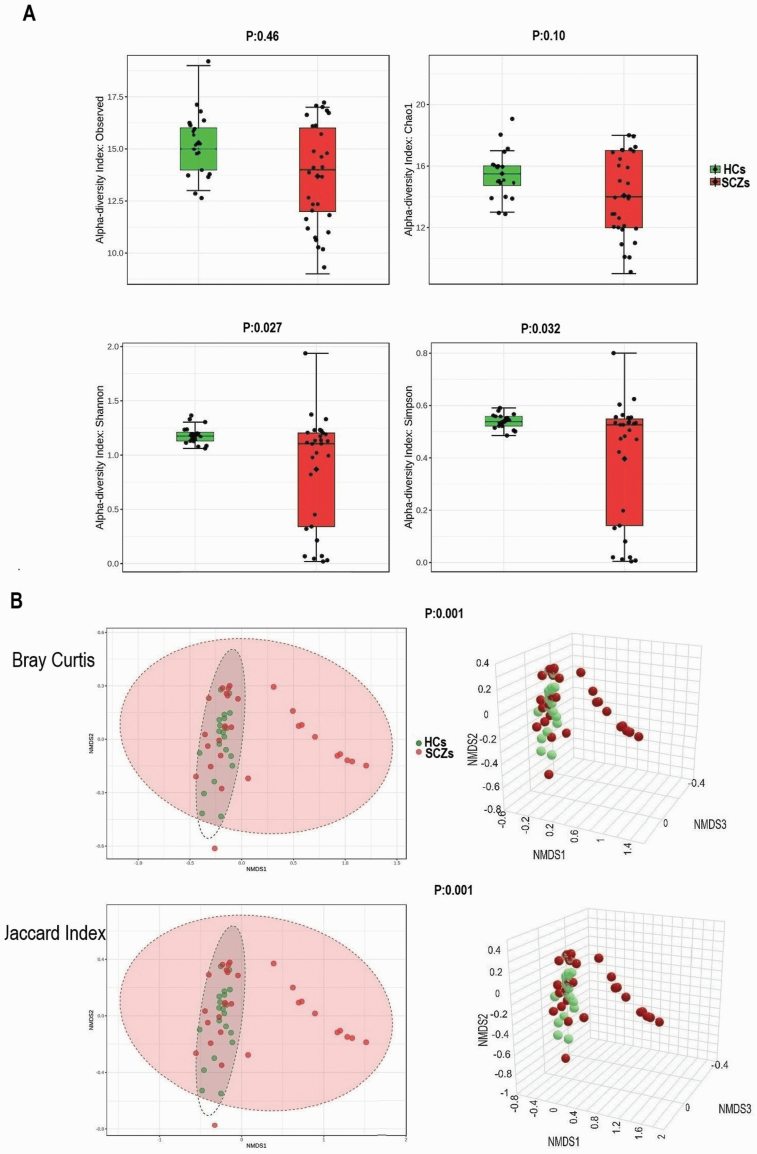
The brain virome richness and diversity and interpersonal variations in HCs and SCZs. (A) Boxplot of alpha diversity of Observed and Chao1, Shannon and Simpson’s indices reflect the abundance and diversity of viral species in the samples. (B) PERMANOVA-validated nonmetric multidimensional scaling (NMDS) beta variety depicted with Bray-Curtis and Jaccard index distances. HCs, healthy controls; SCZs, schizophrenia.

The richness of the brain virome varied considerably between HCs and SCZs. The HCs demonstrated greater viral diversity than the SCZs (alpha diversity: observed index: *P* = .046; Shannon index: *P* = .027; and Simpson’s index: *P* = .032) (figure 2A). Bray-Curtis and the Jaccard index distances based on NMDS revealed interpersonal differences between HCs and SCZs (Bray-Curtis and PERMANOVA: *P* = . 001; Jaccard index and PERMANOVA: *P* = .001) (figure 2B).

### Taxonomic Differences of Brain Virome Between HCs and SCZs

**Fig. 3.**
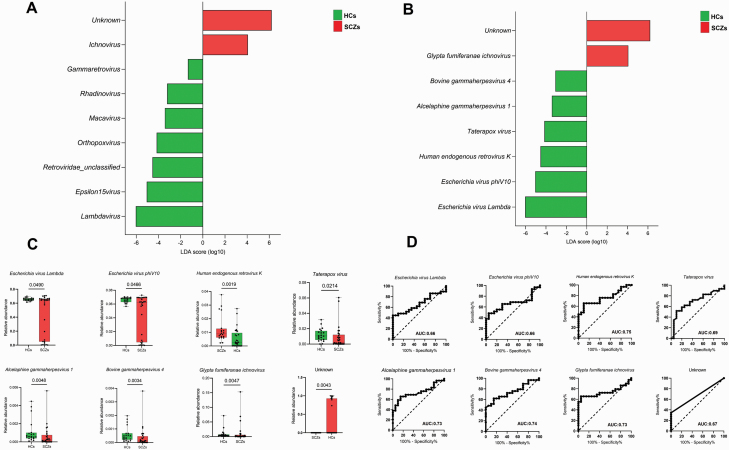
Brain viral signatures significantly different between in HCs and SCZs (A), (B) linear discriminant analysis effect size analysis (LEfSe) identified the most differentially abundant viral genera and species between HCs and SCZs, respectively (*P* < 0.05; LDA score 2). HC-associated viral genera or species are indicated with negative LDA scores (Left), and SCZ-associated viral genera or species indicated with positive LDA scores (Right). (B) Mann-Whitney test for each (*P* < 0.05). (D) Receiver operating characteristic (ROC) analysis, the area under the curve (AUC) values obtained by ROC analysis show the predictive power of the individual viral species differs between HCs and SCZs (AUC; 95% CI). HCs, healthy controls; SCZs, schizophrenia.

**Fig. 4.**
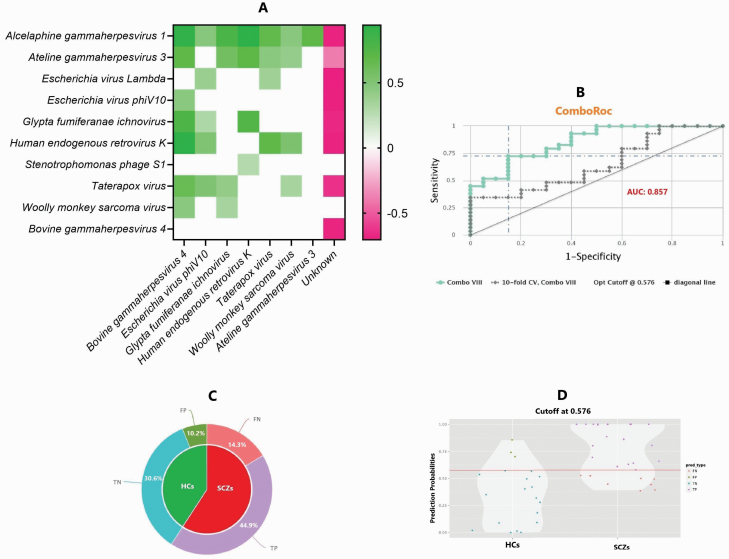
Evaluation of the predictive power of viral signatures identified between healthy controls (HCs) and schizophrenia individuals (SCZs). (A) The heatmap represents the co-occurrence of viral signatures using Spearman correlation analysis. (B) The CombiROC curve combines multiple biomarkers and shows their combine AUC power to distinguish between the 2 compared classes. (C) The pie charts provide an overview of the model’s predictive performance by illustrating the proportions of false negatives (FN), false positives (FP), true negatives (TN), and true positives (TP). (D) The violin plot displays the probability density of data for HCs and SCZs groups, based on the optimal cutoff determined from the corresponding ROC curve. AUC, area under the curve; ROC, receiver operating characteristic.

LEfSe identified distinct genera between HCs and SCZs (figure 3A). HCs exhibited a greater abundance of specific viruses, including *Escherichia virus Lambda*, *Escherichia virus phiV10*, *Human endogenous retrovirus K*, *Taterapox virus*, *Alcelaphine gammaherpesvirus 1*, and *Bovine gammaherpesvirus 4*, while SCZs had a considerably higher abundance of *Glypta fumiferanae ichnovirus* and an unknown virus (*P* < .05; LDA score 2) (figure 3B). The Mann-Whitney test for each of the identified viral signatures displayed in figure 3C yielded *P*-values <.05. ROC analysis results (figure 4D) indicated that certain viral signatures, such as *Human endogenous retrovirus K*, *Bovine gammaherpesvirus 4*, *Alcelaphine gammaherpesvirus 1*, and *Glypta fumiferanae ichnovirus*, provided area under the curve (AUC) values of 0.73–0.75 (AUC; 95% CI, *P* < .05), whereas the remaining viral signatures, including *Taterapox virus*, unknown virus, *Escherichia virus Lambda*, and *Escherichia virus phiV10*, exhibited AUC values ranging from 0.66 to 0.69 (AUC; 95% CI, *P* < .05).

To determine if there is an association between the significant viruses as collaborative members (co-occurrence of viruses) in schizophrenia pathogenesis, spearman correlation was tested and revealed that the viruses enriched in the HCs exhibited a positive correlation with each other (rho = 0.29–0.93, *P* = .05), but a negative correlation with unknown viruses enriched in SCZs (figure 3A).The CombiROC curve depicted in figure 4B has an overall value of 0.857, indicating high accuracy in the identification of viral signatures that distinguish HCs from SCZs. 30.6% of the entire cohort were true negatives and 10.2% were false positives for HCs, whereas 44.9% of the cohort were true positives and 14.3% were false negatives for SCZs (figure 4C). In addition, the violin plot of the CombiROC analysis depicted the distinction between HCs and SCZs based on the viral signatures identified by LEfSe, including true negative, true positive, false negative, and false positive individuals (figure 4D).

## Discussion

The results of this study provide evidence for the presence of a virome profile in the BA46 of both HCs and SCZs. The study analyzed 49 samples, including 20 HCs and 29 SCZs, and identified 91 virus types assigned to 9 phyla, 10 classes, 10 orders, 13 families, 19 genera, and 30 species. Remarkably, *Escherichia virus Lambda* and *Escherichia virus phiV10*, which infect *Escherichia coli*, were found to be significantly more prevalent in the brains of HCs compared with those of individuals with schizophrenia*. Escherichia virus Lambda*, commonly referred to as the Lambda phage, exhibits dual behavior in *E. coli.* It can cause lytic infections, where it replicates within the bacterium, ultimately leading to its destruction. Alternatively, it can establish a lysogenic lifecycle, integrating its genetic material into the host’s genome and remaining dormant for an extended period. This observation raises the possibility that *Escherichia virus Lambda* may influence the composition and diversity of *E. coli*, potentially holding implications for schizophrenia.^32–35^ Previous investigations have observed relatively high levels of antibodies against *E. coli* in the blood serum of individuals with schizophrenia.^36,37^ This may indicate an immune response to *E. coli* in individuals affected by schizophrenia, moreover, increased levels of *E. coli* have been detected in the gut microbiome of both first-episode drug-naive and chronic antipsychotic-treated schizophrenia patients when compared with HCs.^38^ This finding suggests a potential dysregulation of the gut-brain axis and implies that *E. coli* may play a role in the pathogenesis of schizophrenia. Notably, although these pieces of evidence and this study’s findings suggest a possible association between the *Escherichia virus Lambda*, *E. coli*, and schizophrenia, additional research is necessary to determine the precise mechanisms and causal relationships involved.

This study found that *Human endogenous retroviruses* (HERVs) were more abundant in the brains of HCs compared with SCZs. HERVs are remnants of ancient retroviral infections that have become integrated into the human genome over evolutionary time.^39,40^ While much of the research surrounding HERVs is focused on their potential associations with disease,^40^ there is also growing interest in understanding their potential roles in normal physiology, including brain function. Some studies suggest that HERVs may have regulatory functions in the human genome, influencing gene expression and the regulation of cellular processes relevant to the brain. Additionally, HERVs may have played a role in the evolution of the human brain by providing a source of genetic novelty and regulatory elements that shaped brain development and function.^39,41^ Furthermore, HERVs can elicit immune responses, and some HERV proteins have been found to have immunomodulatory properties.^42^ Given the involvement of immune system dysregulation in various neurological disorders, HERVs may influence the immune response in the brain and contribute to the modulation of neuroinflammation and neuroimmune interactions. This study found that certain *gammaherpesviruses*, such as *alcelaphine gammaherpesvirus 1* and *bovine gammaherpesvirus 4*, were more prevalent in the brain of HCs compared with SCZs. gammaherpesviruses are a subgroup of the *Herpesviridae* family and can establish lifelong latent infections in their natural hosts. While some *gammaherpesviruses* are known pathogens, evidence suggests that not all are pathogenic, and some may even have a protective role. For example, a study showed that *murid herpesvirus 4* (MuHV-4) (which belongs to *gammaherpesviruses*) inhibited the development of asthma triggered by house dust mites by modulating the lung’s innate immune system. This occurred through the replacement of resident alveolar macrophages with regulatory monocytes, which suppressed the specific immune response involved in allergies.^43^ However, it is important to consider that gammaherpesviral infections can have complex effects and may lead to diseases under certain circumstances. The association between asthma and schizophrenia was also observed in longitudinal studies, indicating an increased risk of schizophrenia among individuals with asthma.^44^ Similarly, childhood asthma hospitalizations were associated with higher rates of bipolar disorder and schizophrenia spectrum disorders.^45^ While there is emerging evidence linking viral signatures to schizophrenia, their role needs to be evaluated alongside genetic, environmental, and neurobiological factors. Genetic susceptibility plays a significant role in schizophrenia and understanding how viral infections interact with specific genes is crucial. Environmental factors, such as prenatal infections and stressors, also contribute to the disorder, and investigating their interaction with viral signatures is essential. Additionally, exploring the impact of viruses on neurobiological factors can shed light on the mechanisms underlying schizophrenia. Advancing our understanding requires interdisciplinary research, longitudinal studies, and large-scale population studies. By integrating data from various sources, we can gain a comprehensive understanding of the complex interplay between viral signatures and schizophrenia, potentially leading to targeted prevention and treatment strategies. To improve our understanding of the role of the virome in schizophrenia and other brain diseases, future research should resolve several limitations identified in this study. These limitations include the use of SRA metagenomics data with limitation in detailed clinical information and a relatively small sample size, which compromises the reliability of the results. Future studies should include larger sample sizes and collect comprehensive clinical data, controlling for potential confounding factors by considering variables such as age, gender, disease duration, and medication use. Due to the limited availability of human brain tissue samples, which are typically obtained postmortem, studying the brain virome is difficult, comparatively, physiological sample type such as saliva, feces, and blood are more accessible in living organisms. The use of appropriate animal models that simulate human brain maladies and the collection of brain tissue samples from these models is a potential solution for overcoming this limitation. In addition, it is necessary to investigate various brain regions and viral components that may not be adequately preserved during DNA and RNA extraction, to effectively preserve these components for accurate analysis, specialized stabilization procedures may be required. As observed in this study, SCZs have a high abundance of unknown viruses whose types could not be determined, it is recommended to compare various virome databases during the bioinformatics analysis to identify unknown viral species in brain tissues. Future research that addresses these obstacles and limitations will increase our understanding of the role of the brain virome in schizophrenia and other neuropsychiatric diseases.

In conclusion, this study highlights the emerging field of the brain virome and its potential relevance to schizophrenia. The presence of a brain virome suggests a complex relationship between viral communities and the development of schizophrenia. Imbalances in the brain virome might play a role in the development of this psychiatric disorder. One important implication is the possibility of identifying biomarkers that could aid in early diagnosis. By studying the composition and activity of viral communities in the brain, researchers may discover specific viral markers that can serve as early indicators of schizophrenia-related processes. Early detection may facilitate timely intervention, improving clinical outcomes and the quality of life for individuals with schizophrenia. However, further research is needed to fully understand the role of the brain virome in the onset and progression of schizophrenia. Future studies should focus on larger sample sizes, comprehensive clinical information, and the examination of different brain regions and viral components. Additionally, comparing various virome databases can validate the presence and significance of the virome composition in brain tissues.

## Supplementary Material

sgad029_suppl_Supplementary_Tables_S1

## Data Availability

The metagenomic data are available under NCBI BioProject: PRJNA422380 permission based on free and unrestricted access to all of the data under Nucleotide Sequence Database Policies Science 298 (5597): 1333, November 15, 2002.
